# Concurrent Validity of Systematic Nutritional Clinical Assessment in Hospitalized Adults: Comparative Analysis of Patients With and Without Excess Body Weight

**DOI:** 10.1111/jhn.70342

**Published:** 2026-10-08

**Authors:** Marina da Silva Guedes, Larissa Farinha Maffini, Isabela Faller Prade, Teodoro Baptista da Silva Veronese, Ana Clara Vital Batista, Monalisa Reis Arruda, Paulo Cesar Tanuri Bento Junior, Adriana Cruz Rios Behrens, André Castro Lyra, Gabriela Corrêa Souza, Lucivalda Pereira Magalhães de Oliveira, Rosangela Passos de Jesus, Valesca Dall'Alba

**Affiliations:** ^1^ Department of Nutrition, School of Medicine Federal University of Rio Grande do Sul Porto Alegre Brazil; ^2^ Postgraduate Program in Endocrinology, School of Medicine Federal University of Rio Grande do Sul Porto Alegre Brazil; ^3^ Postgraduate Program in Food, Nutrition, and Health, School of Nutrition Federal University of Bahia Salvador Brazil; ^4^ Nutrition Service Hospital Aristides Maltez Salvador Brazil; ^5^ Postgraduate Program in Medicine and Health (PPGMS) Federal University of Bahia Salvador Brazil; ^6^ Nutrition and Dietetics Service Hospital de Clínicas de Porto Alegre Porto Alegre Brazil; ^7^ Postgraduate Program in Food, Nutrition, and Health, School of Medicine Federal University of Rio Grande do Sul Porto Alegre Brazil; ^8^ Postgraduate Program: Sciences in Gastroenterology and Hepatology, School of Medicine Federal University of Rio Grande do Sul Porto Alegre Brazil

**Keywords:** nutritional assessment, nutritional screening, nutrition‐focused physical examination, obesity, overweight, undernutrition

## Abstract

**Introduction:**

Excess body weight can underestimate undernutrition in hospitalized patients. Performing standardised Nutrition‐Focused Physical Examination may reduce subjectivity. This study aimed to assess the concurrent validity of the Systematic Nutritional Clinical Assessment tool and its ability to identify nutritional depletion in patients with excess body weight.

**Methods:**

This cross‐sectional study included adult patients hospitalized in two Brazilian regions. Within 48 hours of admission, patients underwent anthropometric assessment, handgrip strength, Subjective Global Assessment or Patient‐Generated Subjective Global Assessment, and Systematic Nutritional Clinical Assessment.

**Results:**

A total of 412 patients were included (57.6 ± 14.3 years; 58.7% male), from oncology (29.1%), hepatology (28.6%), cardiology (24.0%), and nephrology (18.2%). Undernutrition frequency was 51.4% by Subjective Global Assessment/Patient‐Generated Subjective Global Assessment and 35.7% by Systematic Nutritional Clinical Assessment. The Systematic Nutritional Clinical Assessment showed moderate agreement with the Subjective Global Assessment (*k* = 0.572; *p* < 0.001), high diagnostic accuracy (AUROC 0.886), 63.7% sensitivity, and 94% specificity. In the subgroup of patients with excess body weight, the post‐test probability for positive results increased from 31.7% to 93.1%. The biceps and triceps compartment stood out for high sensitivity (83.0%), while the dorsum of hands and interosseous muscles stood out for high specificity (87.5%).

**Conclusion:**

The Systematic Nutritional Clinical Assessment is shown to be a reliable instrument for confirming nutritional depletion in patients with and without excess body weight. When a simplified physical examination is required during nutritional assessment, the evaluation of the dorsum of hands and interosseous muscles showed the highest specificity.

## Highlights

- The research evaluated the concurrent validity of the Systematic Nutritional Clinical Assessment (SyNCA) against the Subjective Global Assessment in hospitalized adults, comparing patients with and without excess body weight.
- SyNCA demonstrated high diagnostic accuracy and good specificity, but lower sensitivity, making it a highly reliable tool for confirming undernutrition rather than for initial screening.
- For patients with excess body weight, SyNCA's specificity increased to 98.6%, and the post‐test probability of confirming depletion after a positive result jumped from 31.7% to 93.1%, proving its strong confirmatory power.
- When simplifying the physical exam, inspecting the biceps and triceps provides the highest sensitivity, while evaluating the dorsum of the hands and interosseous muscles yields the highest specificity.

## 1 Introduction

Hospitalized patients are frequently affected by undernutrition [1], with rates ranging between 20% and 50%, reaching as high as 60% in Latin American countries. It is important to emphasize that undernutrition can be underestimated in patients with excess body weight, where adiposity hides muscle hypotrophy, hindering early diagnosis and the implementation of timely interventions [1, 2].

Furthermore, the presence of undernutrition is associated with changes in body composition, reduced functionality, increased risk of falls and mortality, impaired immune response and healing, prolonged length of hospital stay, higher hospital costs, higher readmission rates, and worse clinical prognosis [1, 2, 3, 4, 5].

Regarding the pathophysiological mechanisms leading to undernutrition, two deserve highlighting: in the first, the disease leads to a higher energy demand, resulting in the catabolism of muscle and adipose tissue. In the second, the disease triggers symptoms that affect eating, such as anorexia, diarrhoea, nausea, and vomiting, reducing food intake and inducing tissue catabolism to reconstitute Adenosine Triphosphate (ATP) and meet bodily energy demands [6].

Nutrition‐Focused Physical Examination (NFPE) stands out among the methods used in clinical practice as it is characterized by an evaluation of signs frequently used to detect nutritional deficiencies. Such signs and clinical manifestations are identified through inspection, palpation, and auscultation, and can also reveal organic changes presented in muscle and adipose tissues. With a detailed assessment directed toward the inspection of muscle mass and fat stores, NFPE can enable the evaluation of an individual's functional capacity, becoming an important tool for the nutritional monitoring of hospitalized patients [7].

Although NFPE is a non‐invasive and low‐cost assessment traditionally used in clinical practice, until 2026 there were no standardised protocols for evaluating body compartments through physical examination to reduce the limitations associated with examiner subjectivity [7, 8].

To reduce subjectivity and inter‐examiner variability, a systematized instrument for performing NFPE was proposed, specifically tailored for hospitalized patients with chronic non‐communicable diseases [8]. This study aims to assess the concurrent validity of the Systematic Nutritional Clinical Assessment (SyNCA). Additionally, due to the difficulty of establishing a nutritional diagnosis in patients with excess body weight, this study aims to evaluate the capacity of SyNCA to identify nutritional depletion in this specific population. Finally, in order to provide practicality for the assessment, the study aims to identify which body segment evaluated by SyNCA presents the highest diagnostic accuracy, sensitivity, and specificity in relation to the Subjective Global Assessment (SGA) for the detection of nutritional depletion.

## 2 Materials and Methods

### 2.1 Study Design and Population

This is a multicenter cross‐sectional study with a sample composed of individuals admitted to a university hospital in Southern Brazil and a university hospital complex in Northeastern Brazil, between 2022 and 2025. Patients aged 18 years or older, hospitalized for a maximum of 48 h in the specialties of hepatology, cardiology, nephrology, or oncology, were included. Patients in the Intensive Care Unit (ICU) and pregnant women were not included. Patients with incomplete SyNCA and/or SGA assessments were excluded. This study was reported in accordance with the Strengthening the Reporting of Observational Studies in Epidemiology (STROBE) guidelines.

The institution's ethics committees previously approved the study, and all patients provided written informed consent.

### 2.2 Data Collection

Demographic and clinical history data were collected from medical records. All volunteers had their weight and height measured and were evaluated by anthropometry, the SyNCA, and the SGA or Patient‐Generated Subjective Global Assessment (PG‐SGA) [9].

For patients with ascites and/or peripheral edema, dry weight was estimated by reducing 5%, 10%, or 15% of the current weight, according to the severity of the ascites, with an additional 5% reduction in the presence of lower limb fluid retention [10, 11]. For the classification of excess body weight, Body Mass Index (BMI) was considered. Those with a BMI greater than or equal to 25 kg/m^2^ were classified as having excess body weight [10].

Arm circumference (AC) was measured using an inelastic tape at the midpoint between the acromion and the olecranon [10]. For those with excess body weight, AC was adjusted according to BMI [12]. The cutoff points assumed for low AC were 28 cm for men and 25 cm for women [12].

Calf circumference (CC) was measured with the patient seated and the tape measure positioned at the maximum circumference of the calf [10]. Individuals with calf edema were excluded from CC evaluation. Adjustments to CC were made for patients with BMI > 24.9 kg/m^2^ [13]. The cutoff values assumed for low CC were 34 cm for men and 32 cm for women [13]. AC and CC were not measured in patients presenting anasarca. The Mid‐Arm Muscle Circumference (MAMC) was calculated [14, 15].

Muscle strength assessment was performed using Handgrip Strength (HGS) with a hydraulic dynamometer (Jamar or Saehan, both with a 2 kgf resolution). The test was conducted on the non‐dominant hand with the arm unsupported. The patient was asked to perform three series of maximum voluntary contractions lasting 3 seconds each, with a 30‐second rest between measurements. The mean of the three obtained values was considered for the quantitative analysis of muscle strength [16]. Reference values less than or equal to 27 kgf for males and 16 kgf for females were considered low [17].

The SGA was performed [9]. An SGA classification was identified [A‐well nourished; B‐moderately malnourished (or suspected of being) malnourished; or C‐severely malnourished] to indicate the nutritional status of each patient based on subjective weighting [9]. The PG‐SGA was administered to patients with oncological diseases [18].

The SyNCA was performed according to the proposed form [8]. The evaluation was based on the inspection of the body segments. A score was assigned to each evaluated segment as follows: (0) indicated well‐preserved muscular and adipose tissue, with no prominence of bony structures; (2) indicated slight muscular and adipose tissue depletion, with bony structures slightly apparent but not prominent; (4) indicated moderate depletion of muscular and adipose tissue, with bony structures moderately apparent and slightly prominent, mostly covered with adipose tissue and thinning skin; (6) represented severe cases, characterized by marked depletion of muscle and adipose tissue with bony structures markedly apparent and prominent, mostly covered by thin skin. The final score, Nutritional Depletion Index (NDI), was calculated as the sum of the scores from all inspected segments. An NDI of 0–21 classifies the patient as “No Nutritional Depletion”, 22–42 as “Mild Nutritional Depletion”, 43–63 as “Moderate Nutritional Depletion”, and 64–84 as “Severe Nutritional Depletion” (Supplementary Figure 1) [8].

### 2.3 Statistical Analysis

Data normality was checked graphically (histogram, Q‐Q plot, and boxplot) and by the Shapiro‐Wilk test [19]. Continuous variables are described as mean ± standard deviation (normal distribution) or median (interquartile range) (non‐normal distribution). Categorical variables are presented as absolute and relative frequencies.

To compare the means of continuous variables between groups with and without excess weight, the Student's t‐test (normal distribution) or Mann‐Whitney test (non‐normal distribution) was used. Categorical variables were associated using the Chi‐square test or Fisher's exact test.

For the concurrent validity analysis of the SyNCA, using the SGA as the reference method, the SyNCA categories “Mild,” “Moderate,” and “Severe Nutritional Depletion” were grouped, as were SGA categories “B” and “C.” A diagnostic performance analysis was conducted: the predictive power of the SyNCA tool was evaluated by calculating sensitivity, specificity, positive predictive value (PPV), negative predictive value (NPV), and global accuracy. Cohen's Kappa coefficient was applied to verify the degree of agreement between SyNCA and SGA diagnoses. Additionally, a Receiver Operating Characteristic (ROC) curve was constructed [20].

For Kappa interpretation, the reference values were: < 0 (poor), 0–0.2 (slight), 0.21–0.4 (fair), 0.41–0.6 (moderate), 0.61–0.8 (substantial), and 0.81‐1 (almost perfect) [21]. Cutoff values for sensitivity, specificity, PPV, and NPV were: High (90%–100%), Moderate (80%–89%), and Low (≤ 79%) [22].

To estimate the clinical impact of the test on the probability of undernutrition, the Positive Likelihood Ratio (PLR) and Negative Likelihood Ratio (NLR) were calculated. Pre‐test probability was calculated using the positive cases from the SGA. Post‐test probabilities for positive and negative results were determined using Fagan's Nomogram via a digital calculator [23]. Additionally, DELTA (the difference between post‐test and pre‐test probability) was calculated to quantify the actual diagnostic gain provided by the examination.

Diagnostic accuracy (sensitivity, specificity, PPV, NPV, and Kappa agreement) was analyzed for the tool as a whole and independently for each body segment evaluated by the SyNCA in relation to the SGA. To understand the influence of prior nutritional status on tool performance, all analyses were stratified into two subgroups: patients “with excess body weight” and “without excess body weight.”

A *post hoc* sample size calculation determined that 308 subjects were required to estimate the sensitivity and specificity of SyNCA for diagnosing nutritional depletion, with a 15% confidence interval width. Using the Wald method, the calculation assumed a 95% confidence level, a 51.4% proportion of undernutrition according to the SGA, a sensitivity of 63.7%, and a specificity of 94.0%. This analysis was conducted using the online version of the PSS Health tool [24]. Finally, data were analysed using IBM SPSS Statistics 29.0, and statistical significance was set at 5% for all analyses.

## 3 Results

### 3.1 General Characteristics

A total of 596 patients were initially assessed for eligibility. Of these, 437 individuals provided informed consent to participate in the study. Following data collection, 25 patients were excluded from the analysis due to incomplete SyNCA and/or SGA assessments. Consequently, the final analytical sample comprised 412 participants.

The mean age of the sample was 57.61 ± 14.4 years, and the majority were male (58.7%), self‐declared non‐white (53.3%), had completed high school (37.4%), were married (47.2%), and were retired (50.6%). Regarding admission specialties, the sample consisted of 120 (29.1%) individuals treated for oncological diseases, 118 (28.6%) for hepatic, 99 (24%) for cardiac, and 75 (18.2%) for renal conditions. The admission specialty with the highest frequency in the group of patients without excess weight was hepatology, while cardiology was most prevalent in the group with excess weight. White patients presented a higher frequency of excess body weight (Table 1).

**Table 1:** Sociodemographic profile of the sample of hospitalized patients, stratified by excess weight.

Variable	Total (*n* = 412)	With excess body weight (*n* = 202)	Without excess body weight (*n* = 210)	*p*
Age (years)	57.61 ± 14.4	57.55 ± 12.8	57.68 ± 15.7	0.929^c^
Male sex	242 (58.7%)	112 (55.4%)	130 (61.9%)	0.183^a^
Self‐declared non‐white^d^	196 (53.3%)	85 (46.2%)	111 (60.3%)	**0.035** b
Education level				
No formal education	125 (30.3%)	56 (27.7%)	69 (32.9%)	0.430^a^
Primary school completed	80 (19.4%)	36 (17.8%)	44 (21.0%)	
High school completed	154 (37.4%)	82 (40.6%)	72 (34.3%)	
Higher education completed	53 (12.9%)	28 (13.9%)	25 (11.9%)	
Occupational status^e^				
Employed	103 (25.2%)	58 (29.0%)	45 (21.5%)	0.135^a^
Unemployed	99 (24.2%)	50 (25.0%)	49 (234%)	
Retired	207 (50.6%)	92 (46.0%)	115 (55.0%)	
Marital status^f^				
Single	141 (34.3%)	61 (30.3%)	80 (38.1%)	0.214^a^
Married	194 (47.2%)	104 (51.7%)	90 (42.9%)	
Divorced	40 (9.7%)	21 (10.4%)	19 (9.0%)	
Widowed	36 (8.8%)	15 (7.5%)	21 (10.0%)	
Admission specialty				
Cardiology	99 (24.0%)	69 (34.2%)	30 (14.3%)	< **0.001** a
Hepatology	118 (28.6%)	36 (17.8%)	82 (39.0%)	
Nephrology	75 (18.2%)	35 (17.3%)	40 (19.0%)	
Oncology	120 (29.1%)	62 (30.7%)	58 (27.6%)	

*Note:* Data are presented as mean ± standard deviation and absolute (relative) frequency. Statistical significance was set at 5%.

^a^ Chi‐square test.

^b^ Fisher's exact test.

^c^ Student's *t*‐test.

^d^ Total sample = 368; Sample in the excess weight group = 184; Sample in the group without excess weight = 184.

^e^ Total sample = 409; Sample in the excess weight group = 200; Sample in the group without excess weight = 209.

^f^ Total sample = 411; Sample in the excess weight group = 201; Sample in the group without excess weight = 210.

### 3.2 Nutritional Characteristics

The sample presented a mean BMI of 25.47 ± 5.1 kg/m^2^ and an MAMC of 23.49 ± 3.8 cm. Low HGS was observed in 48.5% (*n* = 189) of the patients, while 51.5% (*n* = 209) had low AC and 59.8% (*n* = 220) had low CC. Patients without excess body weight showed lower measured weight, dry weight, BMI, and MAMC. Furthermore, a higher proportion of patients without excess weight presented reduced HGS and AC. There was no significant difference in CC between the groups (Table 2).

**Table 2:** Nutritional profile of the sample of hospitalized patients, stratified by excess weight.

Variable	Total (*n* = 412)	With excess body weight (*n* = 202)	Without excess body weight (*n* = 210)	*p*
Measured weight (kg)	71.88 ± 15.5	82.65 ± 12.4	61.53 ± 10.1	**< 0.001** b
Dry weight (kg)	70.47 ± 15.7	81.80 ± 12.3	59.58 ± 9.6	**< 0.001** b
BMI (kg/m^2^)	25.47 ± 5.1	29.61 ± 3.5	21.48 ± 2.6	**< 0.001** b
Low AC^d^	209 (51.5%)	78 (39.4%)	131 (63.0%)	< **0.001** a
Low CC^e^	220 (59.8%)	100 (55.6%)	120 (63.8%)	0.106^a^
MAMC (cm)^f^	23.49 ± 3.8	25.22 ± 3.7	21.88 ± 3.0	< **0.001** b
Low HGS^g^	189 (48.5%)	79 (42.5%)	110 (53.9%)	**0.024** a
SGA				
Well‐nourished (A)	200 (48.5%)	138 (68.3%)	62 (29.5%)	**< 0.001** a
Moderately malnourished (B)	169 (41.0%)	58 (28.7%)	111 (52.9%)	
Severely malnourished (C)	43 (10.4%)	6 (3.0%)	37 (17.6%)	
SyNCA				
No nutritional depletion	265 (64.3%)	174 (86.1%)	91 (43.3%)	< **0.001** a
Mild nutritional depletion	94 (22.8%)	23 (11.4%)	71 (33.8%)	
Moderate nutritional depletion	37 (9.0%)	3 (1.5%)	34 (16.2%)	
Severe nutritional depletion	16 (3.9%)	2 (1.0%)	14 (6.7%)	
NDI	13 (30)	4 (14)	26 (30)	< **0.001** c

*Note:* Data are presented as mean ± standard deviation, median (interquartile range), and absolute (relative) frequency. Statistical significance was set at 5%.

Abbreviations: AC, arm circumference; CC, calf circumference; HGS, Handgrip Strength; MAMC, Mid‐Arm Muscle Circumference; NDI, Nutritional Depletion Index; SGA, Subjective Global Assessment; SyNCA, Systematic Nutritional Clinical Assessment.

^a^ Chi‐square test.

^b^ Student's *t*‐test.

^c^ Mann‐Whitney test.

^d^ Total sample = 406; Sample in the excess weight group = 198; Sample in the group without excess weight = 208.

^e^ Total sample = 368; Sample in the excess weight group = 180; Sample in the group without excess weight = 188.

^f^ Total sample = 391; Sample in the excess weight group = 188; Sample in the group without excess weight = 203.

^g^ Total sample = 390; Sample in the excess weight group = 186; Sample in the group without excess weight = 204.

According to the SGA, 48.5% (*n* = 200) of the patients were classified as well‐nourished (SGA A) 41% (*n* = 169) as moderately (or suspected of being) malnourished (SGA B), and 10.4% (*n* = 43) as severely malnourished (SGA C). A higher proportion of patients with excess body weight was considered well‐nourished, while in the group without excess body weight, a larger percentage was considered moderately (or suspected of being) malnourished. According to the SyNCA, the median NDI was 13 (30). In the sample, 64.3% (*n* = 265) were considered to have no nutritional depletion, 22.8% (*n* = 94) had mild nutritional depletion, 9% (*n* = 37) had moderate nutritional depletion, and 3.9% (*n* = 16) had severe nutritional depletion. Among patients with excess body weight, only 1% (*n* = 2) presented severe nutritional depletion, with a median NDI of 4 (14), while this rate was 6.7% (*n* = 14) in those without excess body weight, whose median NDI was 26 (30) (Table 2).

### 3.3 Analysis of Body Segments Evaluated by SyNCA

The body segment that most frequently presented the highest number of assessments with the highest score (6 points) was the biceps and triceps (36, 8.7%), followed by the quadriceps (30, 7.3%) and the deltoid (30, 7.3%). Among individuals with excess body weight, the alteration that presented the highest number of assessments with the highest score was the presence of edema in the lower limbs (4, 2%), followed by alterations in the trapezius and pectoralis major, deltoid, and biceps and triceps, all at 1.5% (*n* = 3) (Supporting Information S1: Table S1).

### 3.4 Synca´s Concurrent Validity

The agreement between the SyNCA and the SGA was moderate for the total sample and all subgroups. The performance of the SyNCA in predicting nutritional depletion was evaluated by the ROC Curve with satisfactory results (AUROC ≥ 0.84). In the global sample, the SyNCA proved to be a tool with high specificity (94%) and weak sensitivity (63.7%). In patients without body excess weight, it presented moderate specificity (83.9%). In those with excess body weight, the specificity of the SyNCA increased to 98.6% while sensitivity decreased to 40.6%. The tool presented a high PPV and low NPV (Table 3).

**Table 3:** Concurrent validity of Systematic Nutritional Clinical Assessment considering the Subjective Global Assessment as reference.

Variable	Total	With excess body weight	Without excess body weight
Kappa (P‐value)	0.572 (< 0.001)	0.461 (< 0.001)	0.506 (< 0.001)
AUROC (95% CI)	0.89 (0.853−0.918)	0.84 (0.774−0.907)	0.86 (0.808−0.911)
Accuracy	0.78	0.80	0.77
Sensitivity, %	63.7%	40.6%	73.6%
Specificity, %	94.0%	98.6%	83.9%
Positive predictive value, %	91.8%	92.9%	91.6%
Negative predictive value, %	70.9%	78.2%	57.1%
Positive likelihood ratio	10.62	29.00	4.57
Negative likelihood ratio	0.39	0.60	0.31

Abbreviations: AUROC, Area Under the Receiver Operating Characteristic; CI, confidence interval.

The application of the SyNCA demonstrated a significant increase in post‐test probability across all strata. Notably, the greatest diagnostic gain (Δ +61.4) occurred in the group with excess body weight, where the probability of detecting nutritional depletion increased from 31.7% to 93.1% after a positive result. In the global sample, the probability of confirming depletion tripled following a positive test (91.8%), whereas for a negative result, the chance of the patient truly not having the condition remains low (29%) (Table 4).

**Table 4:** Diagnostic performance of Systematic Nutritional Clinical Assessment: Pre‐test and post‐test probabilities according to nutritional status.

Sample Result	Pre‐test	Post‐test	Δ (Delta)
Global			
Result (+)	51.40%	91.82%	40.42
Result (−)	51.40%	29.00%	−22.40
With excess body weight			
Result (+)	31.70%	93.08%	61.38
Result (−)	31.70%	21.85%	−9.85
Without excess body weight			
Result (+)	70.50%	91.61%	22.11
Result (−)	70.50%	42.92%	−27.58

### 3.5 Analysis of Diagnostic Performance by Body Segment

By analysing the individual segments that comprise the SyNCA in the global sample, it was observed that the global accuracy ranged between 0.57 (lower limb edema) and 0.78 (deltoid). The compartment that showed the highest agreement was the deltoid (*k* = 0.563), the biceps and triceps stood out for the highest sensitivity (83%) and the intercostal muscles and dorsum of hands and interosseous muscles presented the highest specificity (87.5%). The evaluation of lower limb edema showed the lowest diagnostic performance, with a Kappa agreement of 0.16.

In the group with excess body weight, sensitivity decreased in all segments except for edema. The deltoid maintained the highest accuracy (0.8), the gastrocnemius the best agreement (*k* = 0.505), the biceps and triceps the highest sensitivity (70.3%) and the intercostal muscles and temporal the highest specificity (98.6%). In patients without excess body weight, the deltoid showed the highest agreement (*k* = 0.470), the biceps and triceps presented the highest accuracy (0.78) and sensitivity (88.5%) and edema showed the highest specificity (87.1%), followed by the dorsum of the hands and interosseous muscles (83.9%) (Table 5).

**Table 5:** Performance measure of the body segments evaluated by the Systematic Nutritional Clinical Assessment considering the Subjective Global Assessment as reference.

Variable	Total	With excess body weight	Without excess body weight
Temporalis			
Kappa (*p*‐value)	0.451 (< 0.001)	0.461 (< 0.001)	0.33 (< 0.001)
Accuracy	0.73	0.80	0.71
Sensitivity. %	72.6%	40.6%	76.4%
Specificity. %	72.5%	98.6%	58.1%
Positive predictive value. %	73.7%	92.9%	81.3%
Negative predictive value. %	71.4%	78.2%	50.7%
Masseter			
Kappa (*p*‐value)	0.372 (< 0.001)	0.304 (< 0.001)	0.271 (< 0.001)
Accuracy	0.68	0.72	0.65
Sensitivity, %	58.5%	43.8%	64.9%
Specificity, %	79.0%	84.8%	66.1%
Positive predictive value, %	74.7%	57.1%	82.1%
Negative predictive value, %	64.2%	76.5%	44.1%
Bichat's fat pad			
Kappa (*p*‐value)	0.378 (< 0.001)	0.397 (< 0.001)	0.204 (< 0.001)
Accuracy	0.69	0.76	0.62
Sensitivity, %	58.0%	48.4%	62.2%
Specificity, %	80.0%	88.4%	61.3%
Positive predictive value, %	75.5%	66.0%	79.3%
Negative predictive value, %	64.3%	78.7%	40.4%
Zygomatic arch and mandible lines			
Kappa (*p*‐value)	0.445 (< 0.001)	0.366 (< 0.001)	0.347 (< 0.001)
Accuracy	0.72	0.75	0.70
Sensitivity, %	62.3%	45.3%	69.6%
Specificity, %	82.5%	88.4%	69.4%
Positive predictive value, %	79.0%	64.4%	84.4%
Negative predictive value, %	67.3%	77.7%	48.9%
Trapezius and pectoralis major			
Kappa (*p*‐value)	0.534 (< 0.001)	0.489 (< 0.001)	0.402 (< 0.001)
Accuracy	0.77	0.79	0.75
Sensitivity, %	74.5%	59.4%	81.1%
Specificity, %	79.0%	87.7%	59.7%
Positive predictive value, %	79.0%	69.1%	82.8%
Negative predictive value, %	74.5%	82.3%	56.9%
Intercostal muscles			
Kappa (*p*‐value)	0.499 (< 0.001)	0.445 (< 0.001)	0.332 (< 0.001)
Accuracy	0.75	0.80	0.70
Sensitivity, %	62.7%	39.1%	73.0%
Specificity, %	87.5%	98.6%	62.9%
Positive predictive value, %	84.2%	92.6%	82.4%
Negative predictive value, %	68.9%	77.7%	49.4%
Deltoid			
Kappa (*p*‐value)	0.563 (< 0.001)	0.489 (< 0.001)	0.470 (< 0.001)
Accuracy	0.78	0.79	0.78
Sensitivity, %	75.9%	59.4%	83.1%
Specificity, %	80.5%	87.7%	64.5%
Positive predictive value, %	80.5%	69.1%	84.8%
Negative predictive value, %	75.9%	82.3%	61.5%
Biceps e triceps			
Kappa (P‐value)	0.497 (< 0.001)	0.405 (< 0.001)	0.427 (< 0.001)
Accuracy	0.75	0.72	0.78
Sensitivity, %	83.0%	70.3%	88.5%
Specificity, %	66.5%	73.2%	51.6%
Positive predictive value, %	72.4%	54.9%	81.4%
Negative predictive value, %	78.7%	84.3%	65.3%
Adductor pollicis muscle			
Kappa (P‐value)	0.455 (< 0.001)	0.441 (< 0.001)	0.347 (< 0.001)
Accuracy	0.73	0.77	0.68
Sensitivity, %	61.3%	53.1%	64.9%
Specificity, %	84.5%	88.4%	75.8%
Positive predictive value, %	80.7%	68.0%	86.5%
Negative predictive value, %	67.3%	80.3%	47.5%
Back of the hands and interosseous muscles			
Kappa (P‐value)	0.427 (< 0.001)	0.329 (< 0.001)	0.379 (< 0.001)
Accuracy	0.71	0.74	0.69
Sensitivity, %	55.7%	40.6%	62.2%
Specificity, %	87.5%	89.1%	83.9%
Positive predictive value, %	82.5%	63.4%	90.2%
Negative predictive value, %	65.1%	76.4%	48.1%
Quadriceps			
Kappa (P‐value)	0.518 (< 0.001)	0.451 (< 0.001)	0.409 (< 0.001)
Accuracy	0.76	0.75	0.77
Sensitivity, %	81.6%	68.8%	87.2%
Specificity, %	70.0%	78.3%	51.6%
Positive predictive value, %	74.2%	59.5%	81.1%
Negative predictive value, %	78.2%	84.4%	62.7%
Gastrocnemius			
Kappa (P‐value)	0.549 (< 0.001)	0.505 (< 0.001)	0.396 (< 0.001)
Accuracy	0.77	0.80	0.75
Sensitivity, %	73.6%	54.7%	81.8%
Specificity, %	81.5%	92.0%	58.1%
Positive predictive value, %	80.8%	76.1%	82.3%
Negative predictive value, %	74.4%	81.4%	57.1%
General inspection of the bone structures of the lower limbs			
Kappa (P‐value)	0.526 (< 0.001)	0.446 (< 0.001)	0.396 (< 0.001)
Accuracy	0.76	0.79	0.74
Sensitivity, %	68.4%	45.3%	78.4%
Specificity, %	84.5%	94.2%	62.9%
Positive predictive value, %	82.4%	78.4%	83.5%
Negative predictive value, %	71.6%	78.8%	54.9%
Presence of edema in the lower limbs			
Kappa (P‐value)	0.16 (< 0.001)	0.279 (< 0.001)	0.082 (0.051)
Accuracy	0.57	0.72	0.43
Sensitivity, %	28.8%	37.5%	25.0%
Specificity, %	87.5%	87.7%	87.1%
Positive predictive value, %	70.9%	58.5%	82.8%
Negative predictive value, %	53.7%	75.2%	32.7%

## 4 Discussion

The present study aimed to evaluate the concurrent validity of the SyNCA to identify nutritional depletion in hospitalized adults and older adults in different clinical conditions with and without excess body weight and to identify the body regions of the SyNCA with the highest diagnostic accuracy and agreement with the SGA/PG‐SGA. The patients were stratified by excess body weight, and both groups were homogeneous regarding mean age, sex distribution, education level, occupational status, and marital status, minimizing potential confounding biases in the evaluation of the diagnostic performance of the SyNCA. Patients with excess body weight presented a higher proportion of white individuals and higher education levels. The predominant socioeconomic profile, characterized by a high rate of retirees and low overall education, confers strong external validity to the study, reflecting the population served in the *Sistema Único de Saúde* (SUS) hospitals [25].

The tool identified fewer malnourished individuals than the SGA. This finding is expected in clinical practice, since the SGA is an instrument that, in addition to the physical examination, evaluates the clinical history, naturally identifying more individuals with undernutrition.

The characterization of the nutritional profile elucidates the clinical mechanism underlying the low sensitivity of the SyNCA observed in patients with excess body weight. While the SGA identified some degree of undernutrition (categories B and C) in 31.7% of these individuals, the physical examination of the SyNCA classified only 13.9% of the same group as having some degree of nutritional depletion. This discrepancy can be explained by the fact that excess adipose tissue compromises the identification of classic changes such as muscle hypotrophy and subcutaneous fat depletion, which are the targets of NFPE.

The distribution of admission specialties showed a strong association with the weight profile. A higher proportion of patients with excess weight was observed in cardiology wards, reflecting the metabolic impact of adiposity on cardiovascular diseases. In contrast, hepatology concentrated the highest proportion of individuals without excess body weight, which is clinically consistent with the severe hypercatabolism and sarcopenia present in chronic hepatopathies. This distribution of specialties, which also included oncology and nephrology patients, justifies the high frequency of undernutrition in the global sample, as identified by the SGA.

The underreporting of muscle hypotrophy caused by adiposity was evidenced by anthropometric and muscle function measurements, in which patients without excess body weight presented lower MAMC and a higher proportion of individuals with low HGS and AC. The percentage of patients with low CC was the only variable that did not present a statistically significant difference between patients with and without excess weight. Despite the apparent maintenance of muscle volume, a significant portion of patients with excess body weight presented low HGS. Consequently, for this specific population, the findings demonstrate that the structured physical examination should not be used in isolation, and its association with strength markers, such as dynamometry, is indicated.

In the analysis of the body segments of the SyNCA, it was noted that in all compartments, with the exception of the edema assessment, the majority of patients with excess body weight received a score of “0,” indicating the presence of visually preserved muscle and adipose tissue. The regions where the group with excess body weight presented the highest frequency of null scores (no depletion) were the intercostal muscles, the general inspection of bone structures of the lower limbs, and the dorsum of the hands. In contrast, lower limb edema was consolidated as the only evaluated body segment not influenced by BMI, showing no statistical difference between the stratified groups. The equitable manifestation of fluid overload is linked to the underlying disease, occurring regardless of the patient's adipose reserve.

The segment that presented the greatest nutritional depletion in the global sample and after stratification was the biceps and triceps. The hypothesis is that edema masks the loss of muscle and fat mass in lower limbs and trunk. The arms are much less affected by edema compared to the lower limbs, making nutritional depletion more visible. Furthermore, the arm is a highly accessible and practical region to evaluate in hospitalized patients. The inspection and palpation of the biceps and triceps are areas where evaluators generally have greater familiarity with the assessment, which may justify the higher depletion scores captured by the tool in this location.

In the global evaluation, the tool showed moderate agreement with the SGA and good diagnostic accuracy, evidenced by a high AUROC value, which remained consistent even after stratification by BMI. Regarding intrinsic performance, the accuracy of the SyNCA demonstrated an overall hit rate of 78%, reaching 80% in patients with excess weight and 77% in those without excess body weight.

When analysing sensitivity and specificity, the SyNCA presented low sensitivity and high specificity. This pattern was even more pronounced in the subgroup of patients with excess body weight. For patients without excess body weight, specificity was moderate.

Considering its high capacity to correctly identify individuals without depletion and its lower capacity to detect all affected individuals, the SyNCA acts essentially as a tool with high confirmatory power. In summary, both in the unified and stratified samples, the tool proved to be predominantly specific at the expense of sensitivity, being, therefore, more recommended for the nutritional assessment stage than for screening.

This confirmatory characteristic is corroborated by the analysis of predictive values. The SyNCA demonstrated a high PPV, meaning that, upon a positive result with the tool, the probability of the individual actually having nutritional depletion is very high. This becomes clear in the probability analysis: the pre‐test probability of the global population presenting undernutrition was 51.4%; after applying the SyNCA, in the event of a positive result, this probability jumps to 91.8%. In the group with excess body weight, this jump is even more pronounced, going from 31.7% to 93.1%.

On the other hand, the tool's NPV was low, reflecting the probability of an individual truly not having depletion when the SyNCA result is negative. Due to the low sensitivity, a negative result on the physical examination is not sufficient to rule out the risk. In the global sample, patients identified as having no depletion by the SyNCA still present a 29% post‐test probability of being malnourished. For patients without excess body weight, this uncertainty is even greater. For those with excess weight, the post‐test probability is 21.9%, meaning the risk of a false negative remains significant.

With the objective of identifying which body segments present the highest diagnostic accuracy and agreement with the SGA, lower limb edema was disregarded due to its strong relationship with the underlying disease. Considering clinical applicability in nutritional screening, where sensitivity is prioritized, the biceps and triceps segment appears as the first choice for patients with and without excess body weight. For nutritional assessment, when the patient's nutritional status is not previously known, the intercostal muscles or the dorsum of the hands and interosseous muscles can be used. For patients with excess weight, the most indicated would be the temporal muscle, which has greater practicality, or the intercostal muscles. The dorsum of the hands and interosseous muscles, in turn, are indicated for patients without excess body weight, standing out for their significant ease and practicality, configuring a highly viable strategy in hospitalized patients, who are commonly bedridden and have restricted mobility.

This study presents some limitations that must be considered in the interpretation of the results. First, the methodological design did not allow for the blinding of evaluators. Since the SGA was conducted prior to the SyNCA, prior knowledge of the patient's nutritional status may have introduced an information bias during the application of the new tool.

Second, it is worth highlighting the limitation due to the absence of a gold standard for nutritional diagnosis, which motivated the adoption of the SGA as a reference method. Due to the nature of the SGA, which encompasses clinical history in addition to the physical examination, it is expected to diagnose a higher proportion of malnourished patients than a tool strictly structured around the physical examination. For future investigations, the validation of the SyNCA against body composition assessment methods, such as Dual‐Energy X‐Ray Absorptiometry (DXA), is recommended.

Additionally, the multicenter nature of the research, which included data from four distinct centres, introduces the risk of inter‐rater variability. Although the professionals underwent standardised prior training, the present study did not measure the degree of agreement between examiners, making it impossible to rule out potential differences in the clinical application of the instrument.

## 5 Conclusion

In conclusion, the SyNCA is an essential auxiliary clinical instrument designed to standardise the NFPE, complementing rather than replacing the diagnostic role of the SGA. While its limited sensitivity makes it unsuitable for nutritional screening or ruling out undernutrition, the tool demonstrates good diagnostic accuracy, moderate agreement with the SGA, good specificity, and a strong ability to confirm undernutrition. In patients with excess body weight, the SyNCA appears especially useful for confirming the nutritional diagnosis due to its increased specificity. In this group of patients, its association with strength markers, such as dynamometry, is recommended. When simplification of the physical examination is necessary for screening, inspecting the biceps and triceps provides the highest sensitivity. For nutritional assessment, examine the dorsum of the hands and interosseous muscles (if normal weight or unknown diagnosis) or the temporal muscle (if overweight).

## Author Contributions

G.C. Souza, V. Dall'Alba, R.P. de Jesus, L.P.M. de Oliveira contributed to the conception and design of the research; M.S. Guedes, L.F. Maffini, I.F. Prade, T.B.S. Veronese, A.C.V. Batista, M.R. Arruda, P.C.T.B Junior, A.C.R. Behrens, A.C. Lyra to the acquisition of the data; M.S. Guedes, T.B.S. Veronese contributed to the analysis of the data; M.S. Guedes, T.B.S. Veronese, V. Dall'Alba contributed to the interpretation of the data; M.S. Guedes drafted the manuscript. All authors critically revised the manuscript, agree to be fully accountable for ensuring the integrity and accuracy of the work, and read and approved the final manuscript. Furthermore, all authors acknowledge that they are in agreement with the manuscript and declare that the content has not been published elsewhere.

## Conflicts of Interest

The authors declare no conflicts of interest.

## Supporting information


Supporting File

## Data Availability

Nutrition & Dietetics (NDI).
