# Supplementary material for: Concurrent Validity of Systematic Nutritional Clinical Assessment in Hospitalized Adults: Comparative Analysis of Patients With and Without Excess Body Weight
Source: J Hum Nutr Diet. 2026 Oct 8;39(5):e70342. doi: 10.1111/jhn.70342 (PMC13648220; doi:10.1111/jhn.70342)
Supplement: Supplementary file 1 [file JHN-39-0-s001.docx]

**Supplementary Table 1.** Evaluation of body segments in the sample of hospitalized patients, stratified by excess weight.

| Variable | Total (n=412): n (%) | With excess weight (n=202): n (%) | Without excess weight (n=210): n (%) | P |
| --- | --- | --- | --- | --- |
| Temporalis  0  2  4  6  Masseter  0  2  4  6  Bichat’s fat pad  0  2  4  6  Zygomatic arch and mandible lines  0  2  4  6  Trapezius and pectoralis major  0  2  4  6  Intercostal muscles  0  2  4  6  Deltoid  0  2  4  6  Biceps e triceps  0  2  4  6  Adductor pollicis muscle  0  2  4  6  Back of the hands and interosseous muscles  0  2  4  6  Quadriceps  0  2  4  6  Gastrocnemius  0  2  4  6  General inspection of the bone structures of the lower limbs  0  2  4  6  Presence of edema in the lower limbs  0  2  4  6 | 203 (49.3)  129 (31.3)  57 (13.8)  23 (5.6)  246 (59.7)  110 (26.7)  44 (10.7)  12 (2.9)  249 (60.4)  104 (25.2)  45 (10.9)  14 (3.4)  245 (59.5)  96 (23.3)  49 (11.9)  22 (5.3)  212 (51.5)  92 (22.3)  80 (19.4)  28 (6.8)  254 (61.7)  78 (18.9)  59 (14.3)  21 (5.1)  212 (51.5)  91 (22.1)  79 (19.2)  30 (7.3)  169 (41.0)  127 (30.8)  80 (19.4)  36 (8.7)  251 (60.9)  93 (22.6)  49 (11.9)  19 (4.6)  269 (65.3)  88 (21.4)  38 (9.2)  17 (4.1)  179 (43.4) 129 (31.3)  74 (18.0)  30 (7.3)  219 (53.2)  103 (25.0)  67 (16.3)  23 (5.6)  236 (57.3)  96 (23.3)  57 (13.8)  23 (5.6)  326 (79.1)  57 (13.8)  23 (5.6)  6 (1.5) | 132 (65.3)  57 (28.2)  11 (5.4)  2 (1.0)  153 (75.7)  41 (20.3)  7(3.5)  1 (0.5)  155 (76.7)  41 (20.3)  5 (2.5)  1 (0.5)  157 (77.7)  37 (18.3)  6 (3.0)  2 (1.0)  147 (72.8)  38 (18.8)  14 (6.9)  3 (1.5)  175 (86.6)  19 (9.4)  6 (3.0)  2(1.0)  147 (72.8)  36 (17.8)  16 (7.9)  3 (1.5)  120 (59.4)  56 (27.7)  23(11.4)  3 (1.5)  152 (75.2)  35 (17.3)  14 (6.9)  1 (0.5)  161 (79.7)  31(15.3)  9 (4.5)  1 (0.5)  128 (63.4) 58 (28.7)  14 (6.9)  2 (1.0)  156 (77.2)  30 (14.9)  15 (7.4)  1 (0.5)  165 (81.7)  28(13.9)  8 (4.0)  1(0.5)  161 (79.7)  28 (13.9)  9 (4.5)  4 (2.0) | 71 (33.8)  72 (34.3)  46 (21.9)  21 (10.0)  93 (44.3)  69 (32.9)  37 (17.6)  11 (5.2)  94 (44.8)  63 (30.0)  40 (19.0)  13 (6.2)  88 (41.9)  59 (28.1)  43 (20.5)  20 (9.5)  65 (31.0)  54 (25.7)  66 (31.4)  25 (11.9)  79 (37.6)  59 (28.1)  53 (25.2)  19 (9.0)  65 (31.0)  55 (26.2)  63 (30.0)  27 (12.9)  49 (23.3)  71 (33.8)  57 (27.1)  33 (15.7)  99 (47.1)  58 (27.6)  35 (16.7)  18 (8.6)  108 (51.4)  57 (27.1)  29 (13.8)  16 (7.6)  51 (24.3) 71 (33.8)  60 (28.6)  28 (13.3)  63 (30.0)  73 (34.8)  52 (24.8)  22 (10.5)  71 (33.8)  68 (32.4)  49 (23.3)  22(10.5)  165 (78.6)  29 (13.8)  14 (6.7)  2 (1.0) | **<0.001**^a^  **<0.001**^a^  **<0.001**^a^  **<0.001**^a^  **<0.001**^a^  **<0.001**^a^  **<0.001**^a^  **<0.001**^a^  **<0.001**^a^  **<0.001**^a^  **<0.001**^a^  **<0.001**^a^  **<0.001**^a^  0.644^b^ |

Data are presented as absolute (relative) frequency.

^a^ Chi-square test

^b^ Fisher's exact test

**Supplementary Figure 1.** Study design and data collection. Created using Canva.
